# Death-associated protein kinase 1 as a therapeutic target for Alzheimer's disease

**DOI:** 10.1186/s40035-023-00395-5

**Published:** 2024-01-09

**Authors:** Tao Zhang, Byeong Mo Kim, Tae Ho Lee

**Affiliations:** 1 Fujian Key Laboratory of Translational Research in Cancer and Neurodegenerative Diseases, Institute of Basic Medicine, School of Basic Medical Sciences, Fujian Medical University, 1 Xuefu North Road, Fuzhou, 350122 Fujian China; 2 Research Center for New Drug Development, AgingTarget Inc., 10F Ace Cheonggye Tower, 53, Seonggogae-Ro, Uiwang-Si, 16006 Gyeonggi-Do Korea

**Keywords:** Death-associated protein kinase 1, Alzheimer’s disease, Tau, Amyloid-β, Neuronal cell death, Therapeutic target

## Abstract

Alzheimer’s disease (AD) is the most prevalent form of dementia in the elderly and represents a major clinical challenge in the ageing society. Neuropathological hallmarks of AD include neurofibrillary tangles composed of hyperphosphorylated tau, senile plaques derived from the deposition of amyloid-β (Aβ) peptides, brain atrophy induced by neuronal loss, and synaptic dysfunctions. Death-associated protein kinase 1 (DAPK1) is ubiquitously expressed in the central nervous system. Dysregulation of DAPK1 has been shown to contribute to various neurological diseases including AD, ischemic stroke and Parkinson’s disease (PD). We have established an upstream effect of DAPK1 on Aβ and tau pathologies and neuronal apoptosis through kinase-mediated protein phosphorylation, supporting a causal role of DAPK1 in the pathophysiology of AD. In this review, we summarize current knowledge about how DAPK1 is involved in various AD pathological changes including tau hyperphosphorylation, Aβ deposition, neuronal cell death and synaptic degeneration. The underlying molecular mechanisms of DAPK1 dysregulation in AD are discussed. We also review the recent progress regarding the development of novel DAPK1 modulators and their potential applications in AD intervention. These findings substantiate DAPK1 as a novel therapeutic target for the development of multifunctional disease-modifying treatments for AD and other neurological disorders.

## Background

Alzheimer’s disease (AD) is the most prevalent chronic neurodegenerative disorder. According to the newest statistics, more than 55 million people worldwide are suffering from AD or related dementia, making AD and other dementias the leading cause of disabilities in the elderly [1]. As of 2019, the estimated healthcare cost among people with dementia was more than $590 billion, and this cost is projected to reach $1.6 trillion by 2050 [2]. AD is characterized pathologically by the formation of numerous intracellular neurofibrillary tangles (NFTs) composed of highly phosphorylated tau proteins and extracellular senile plaques composed of amyloid-β (Aβ) peptides. In addition to NFTs and amyloid plaques, oxidative stress, neuroinflammation and synaptic dysfunction are commonly observed during disease progression, and contribute to cell death, breakdown of the blood–brain barrier (BBB) and impairment of neural circuits in the brain. In particular, neuronal loss is a prominent pathological feature in AD, and has been associated with both structural and functional changes in brain regions involved in learning and memory [3].

Tau is a microtubule-associated protein that plays a pivotal role in regulating the microtubule dynamics. The interaction between tau and microtubules is affected by the phosphorylation status of tau in neurons, which is controlled by a host of kinases and phosphatases [4, 5]. The pathological phosphorylation of tau in AD not only drives the dissociation of tau from microtubules, but also affects the localization and solubility of tau in neurons [6–8], thus leading to microtubule disruption and the formation of filamentous paired helical filaments in the AD brain [9–11]. The spatiotemporal accumulation of NFTs in the brain is significantly correlated with disease progression and the degree of cognitive impairment in AD patients [12–14], suggesting a central role of tau hyperphosphorylation in neurodegeneration.

The aggregation of Aβ, a product from amyloid precursor protein (APP) proteolysis, forms soluble oligomers and insoluble fibrils in the brain. The accumulation of Aβ species in the cerebrospinal fluid (CSF) and brain parenchyma is an early pathological feature of AD and has long been considered as an upstream trigger of various pathological changes in disease progression [15]. There are two distinct proteolytic pathways of APP, the non-amyloidogenic processing which involves sequential cleavage of APP by α- and γ-secretases, and the amyloidogenic processing which is mediated by β- and γ-secretases [16–20]. The main products of the amyloidogenic pathway are Aβ40 and Aβ42 [21–23]. The generation of Aβ is regulated by both the function of secretases and the expression of APP [24, 25]. For example, pathogenic mutations in APP and presenilin-1 (PS1) in familial AD strongly promote Aβ production and increase the Aβ42/Aβ40 ratio [26–30]. Besides, post-translational modifications of APP such as phosphorylation, glycosylation and sumoylation also have a direct impact on Aβ generation [31–33].

DAPK1, a serine/threonine (Ser/Thr) protein kinase, was initially identified from an antisense cDNA expression library of HeLa cells as a potential mediator of interferon-γ-induced cell death [34]. In subsequent studies, DAPK1 was shown to play a critical role in stimulus-triggered apoptosis, autophagy, and anoikis-like cell death. The pro-apoptotic function of DAPK1 is closely associated with the pathogenesis of cancer and neurodegenerative diseases [35]. In particular, genetic variations in *DAPK1* have been shown to be associated with late-onset AD (LOAD) in different populations [36–38]. Furthermore, the high abundance of DAPK1 in the cortex and hippocampus also highlights a central role of DAPK1 in regulating neuronal functions [39].

In this review, we discuss the role of DAPK1 and its implications in the pathophysiology of brain diseases, with a particular focus on AD. We also discuss strategies and translational potential for attenuating AD-related neuropathologies through pharmacological or genetic targeting of DAPK1.

## Structural and functional properties of DAPK1

### The DAPK family

**Fig. 1:**
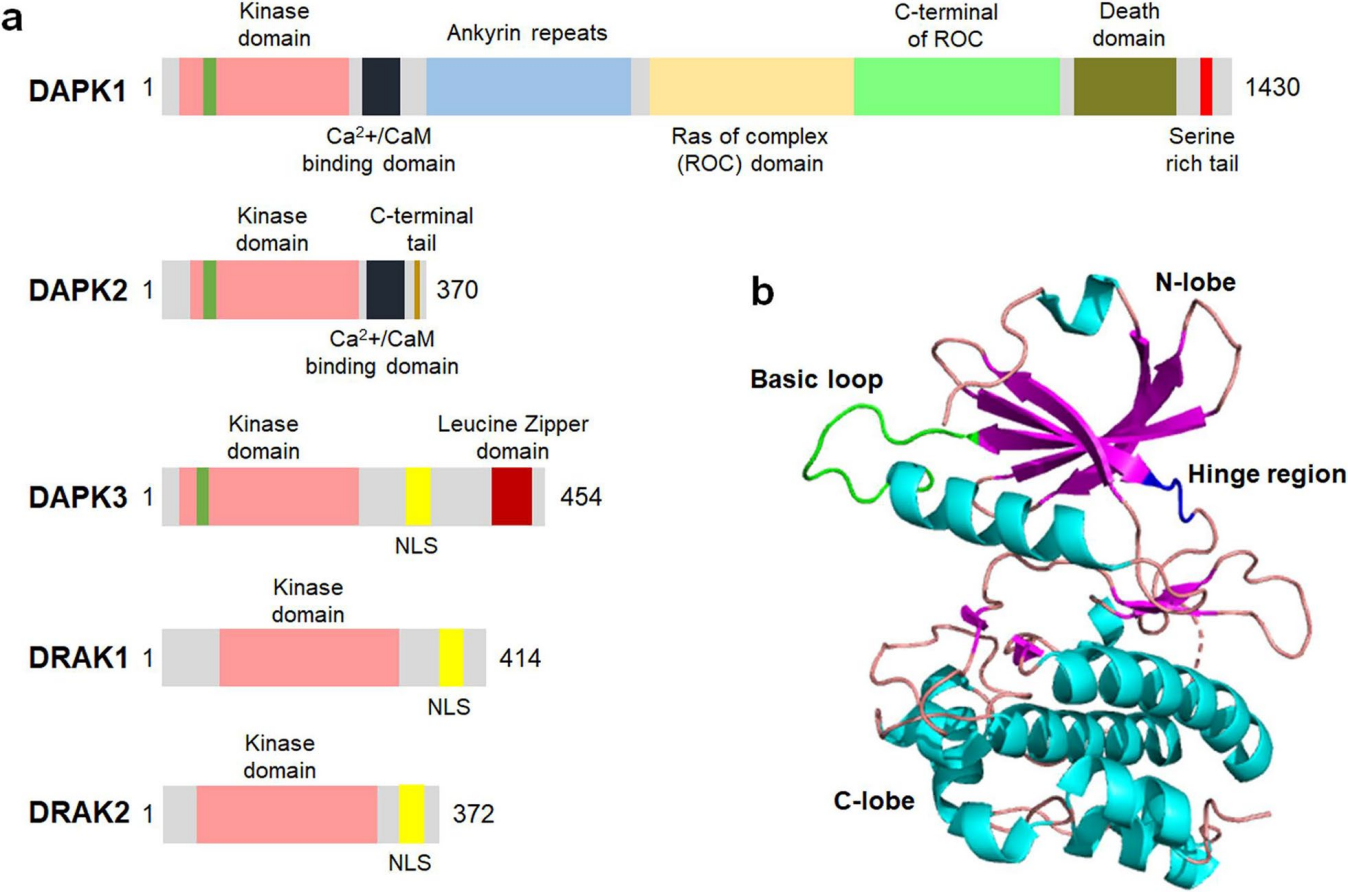
Structures of the DAPK family members. **a** The protein domains of DAPK1, DAPK2, DAPK3, DRAK1 and DRAK2. NLS, nuclear localization signal. **b** A crystal structure of the kinase domain of DAPK1 (Protein Data Bank ID 8IE5). Note that the ligand (oxyresveratrol) has been removed for demonstration purpose

The human DAPK family consists of five kinase members: DAPK1, DAPK1-related protein 1 (DRP-1 or DAPK2), zipper-interacting protein kinase (ZIPK or DAPK3), DAPK1-related apoptosis-inducing protein kinase 1 (DRAK1), and DRAK2 (Fig. 1a). Among these family members, DAPK2 and DAPK3 are highly homologous to DAPK1 in the catalytic domains, showing 80% and 83% identity at the amino acid level, respectively [40]. However, the kinase domains of human DRAK1 and DRAK2 share only 48% and 51% sequence homology with the catalytic domain of DAPK1 [41]. The extra-catalytic domains of DAPK family members vary greatly in size and structure, indicating distinct regulatory mechanisms, subcellular localizations and biological functions of these kinases. For instance, DAPK1 and DAPK2 are mainly found in the cytoplasm, and DAPK3 can be detected in both the cytoplasm and the nucleus [42], while both DRAK1 and DRAK2 are exclusively localized to the cell nucleus [43]. In addition to the subcellular distribution, the DAPK family members also show different tissue expression profiles in the human body. For example, the expression of DAPK1 and DAPK3 has low tissue specificity, while DAPK2 is abundantly expressed in the bone marrow [44].

### DAPK1 structure

DAPK1 is a multidomain protein with distinct functional motifs involved in the modulation of catalytic activity, substrate binding, protein stability and localization of DAPK1. As shown in Fig. 1a, DAPK1 is composed of a kinase domain (13–267 aa), a Ca^2+^/calmodulin (Ca^2+^/CaM)-binding domain (275–334 aa), an ankyrin repeats domain (378–638 aa), two putative P-loops, a Ras of complex (ROC) domain (667–995 aa), a C-terminal of ROC (COR) domain (995–1288 aa), a death domain (DD, 1300–1398 aa) and a C-terminal serine-rich tail [35]. To date, there is no 3D structure of full-length DAPK1 available in the protein data bank, while the structure of the kinase domain of DAPK1 is well-resolved.

The catalytic domain of DAPK1 is composed of 11 subdomains, including a β-sheet-rich N-lobe and an α-helix-rich C-lobe connected by a short hinge region (Fig. 1b). The conserved lysine (K42) in this domain is vital for the kinase activity as it is inside the ATP-binding loop. The catalytic activity of DAPK1 is abolished by substituting the lysine residue with an alanine residue (K42A) [45]. The basic loop region (45–56 aa) containing mostly positively charged residues in the kinase domain is a characteristic motif of the DAPK family as it is responsible for the homo- and hetero-dimerization of DAPK, which is critical for the modulation of kinase activity [46]. In addition to a direct regulation of catalytic activity, the kinase domain also affects the protein stability of DAPK1 by interacting with the chaperone heat shock protein 90 (HSP90) through the amino terminal lobe (αC-β4 loop) [47, 48]. It has been suggested that the enriched neutral and positively charged residues in this subdomain are responsible for the interaction between DAPK1 and HSP90 [48], which further contributes to the maturation and stabilization of cellular DAPK1. The catalytic activity of DAPK1 is also regulated by the Ca^2+^/CaM–autoregulatory domain, which serves as a pseudo-substrate and occupies the catalytic cleft in the absence of CaM, thereby preventing the activation of DAPK1. In addition, the enzymatic activity of DAPK1 is regulated through phosphorylation at several sites that are located within the Ca^2+^/CaM-autoregulatory domain. For example, the autophosphorylation of Ser308 of DAPK1 has an inhibitory effect on its kinase activity [49]. Full activation of DAPK1 requires both dephosphorylation of Ser308 and binding of the Ca^2+^-activated CaM to the autoregulatory domain [50]. The interaction with CaM can release the catalytic cleft of DAPK1, and the dephosphorylation of Ser308 increases the affinity for CaM, thereby promoting the catalytic activity of DAPK1 even at low CaM levels [50].

The ankyrin repeats domain, made up of eight linearly aligned motifs with 33 amino acid residues, is a protein–protein interaction domain facilitating communication with other proteins. This domain is involved in the interaction between DAPK1 and actin filaments, which is pivotal for the correct cellular localization of DAPK1 [51]. Additionally, several ubiquitin ligases such as DAPK-interacting protein 1 (DIP-1) and carboxyl terminal of HSP70-interacting protein have been reported to interact with DAPK1 via the ankyrin repeats domain, thus regulating the proteasomal degradation of DAPK1 in cells [52, 53]. Furthermore, it has been suggested that the phosphorylation of Tyr491/492 residues in the fourth ankyrin repeat by Src kinase inhibits the catalytic activity of DAPK1 [54].

The ROC and COR domains of DAPK1 regulate the kinase activity of DAPK1 by functioning as a GTPase. DAPK1 binds to GTP via the P-loop motif in the ROC domain and hydrolyzes GTP, resulting in conformational changes in the kinase and a reduction in inhibitory Ser308 phosphorylation [55, 56]. Apart from the intrinsic GTPase activity, the ROC-COR domain is able to interact with the phospho-Ser/Thr-directed peptidyl prolyl isomerase 1 (Pin1) via the cytoskeleton-binding region, leading to suppression of the activity of Pin1 that is critical for regulating the function of various phosphoproteins by isomerizing the phospho-Ser/Thr-Pro motif [57, 58].

The death domain of DAPK1 also actively regulates the kinase activity and the degradation of DAPK1, and participates in protein–protein interactions important for cellular apoptosis and energy metabolism. For instance, the extracellular-signal-regulated kinase (ERK) binds to the death domain and phosphorylates the Ser735 residue of DAPK1, which enhances the kinase activity of DAPK1 in vitro and in vivo [59]. DAPK1 is able to interact with Kelch-like family member 20 and tuberous sclerosis 2 protein via the death domain, resulting in the proteasome- and lysosome-mediated degradation of DAPK1, respectively [60, 61]. Interestingly, DAPK1 has been reported to interact with and activate the pyruvate kinase isoform M2 in cancer cells in a kinase activity-independent manner. This interaction alters cellular metabolism by increasing the glycolytic rate and the generation of lactate in cells [62]. The serine-rich tail in the C-terminus acts as a negative regulator of DAPK1 activity as the deletion of this domain augments the pro-apoptotic function of DAPK1 [63].

In addition to the K42A mutant which shows diminished kinase activity, several other DAPK1 mutants have also been developed for research purposes. The ΔCaM, a mutant lacking the Ca^2+^/CaM-binding domain, and the phosphoablative S308A mutant, in which serine 308 is replaced by an alanine residue, are examples of constitutively active forms of DAPK1 with elevated catalytic activity [64]. The ΔCyto, a mutant lacking the cytoskeleton-binding region, shows altered colocalization with actin filaments and thus affects the cellular function of DAPK1 [64].

## DAPK1 and AD

Protein kinases play an important role in the pathogenesis of neurodegenerative diseases [65, 66]. The activities of a variety of neuronal enzymes, receptors, and ion channels are regulated by their phosphorylation status [67, 68]. The spatiotemporal expression of DAPK1 in the brain indicates an essential role of this kinase in the regulation of neuronal functions. Further genetic analyses and animal studies have established that DAPK1 is indeed involved in the pathogenesis of a variety of neurodegenerative diseases. Herein, we focus on the role of DAPK1 in AD based on the research from our group and others, in order to present a comprehensive picture of how DAPK1 contributes to the pathophysiology of AD as well as the therapeutics targeting DAPK1.

### Expression of DAPK1 in the brain

Although DAPK1 is ubiquitously present in different tissues and cell types, it is most abundantly expressed in the brain and lung tissues according to a Northern blotting analysis in rats [39]. In particular, the spatiotemporal expression of DAPK1 in the brain appears to be associated with neurodevelopment and neurogenesis. The temporal analysis of DAPK1 mRNA expression in rat brains revealed that the expression of DAPK1 mRNA is detectable at embryonic day 13 (E13) and further increases to a peak at approximately E20. However, the DAPK1 mRNA level gradually declines after birth [39]. The spatial determination of DAPK1 expression by in situ hybridization shows that DAPK1 mRNA can be extensively found in both proliferative and postmitotic regions within the cerebral cortex, hippocampus, and cerebellum during embryonic development and the neonatal stage [39]. These regions undergo widespread programmed cell death in embryonic neurogenesis [69, 70]. At postnatal and adult stages, the cerebral cortex and cerebellum both manifest a significant decline in DAPK1 mRNA expression, while DAPK1 expression in the hippocampus is maintained at high levels particularly in CA1, CA2 and the dentate gyrus [39, 71]. Besides, neuronal cells have higher DAPK1 expression than glial cells in the brain under physiological conditions. Thus, the widespread expression of DAPK1 during brain development and confined expression in mature neuronal populations in adults suggest that DAPK1 may not only participate in developmental neuronal death, but also play a role in regulating neuronal activity and synaptic functions. Indeed, it has been reported that DAPK1 is substantially enriched in hippocampal synapses and has complex interactions with numerous synaptic proteins [72, 73], which underlies the pathological influence of DAPK1 dysregulation on brain functions in neurological disorders such as AD.

### Dysregulation of DAPK1 expression or function in neurological diseases

As a stress-responsive protein kinase, the dysregulation of DAPK1 expression or its activity has been implicated in a variety of neurological disorders. In Table 1, we briefly summarize how DAPK1 is dysregulated in common neurological diseases such as ischemic stroke, PD, AD, epilepsy and traumatic brain injury (TBI). For instance, cerebral ischemia not only leads to DAPK1 activation, but also induces aberrant interactions between DAPK1 and other proteins such as p53 and GluN2B, thereby leading to neuronal loss and synaptic dysfunction [73, 74]. The activation of DAPK1 in ischemia is likely mediated by the *N*-methyl-*D*-aspartate receptor (NMDAR)-induced Ca^2+^ influx and the subsequent dephosphorylation at Ser308 of DAPK1 by the calcineurin phosphatase [75]. Another common disease with Ca^2+^ signaling disorder is epilepsy. Interestingly, our research demonstrated that DAPK1 dysregulation in epilepsy is model-dependent, as convulsive dosing of pentylenetetrazol (PTZ) immediately activates the DAPK1 function in the mouse brain without upregulating its expression, while chronic exposure to PTZ at a low dose in the kindling model increased both the protein level and the activity of DAPK1 in the mouse brain [76]. Further, our study showed that kainic acid (KA) promotes DAPK1 activity by upregulating the ERK-mediated Ser735 phosphorylation in a mouse epilepsy model [77].

**Table 1:** A summary of DAPK1 dysregulation in common neurological diseases

Disease	Model/sample	Changes in DAPK1	Pathological associations	References
Brain ischemia	Middle cerebral artery occlusion followed by reperfusion (transient ischemia)	Immediate down-regulation of *DAPK1* mRNA in the ischemic core and up-regulation in the medial striatum; increased cleavage of DAPK1; reduction of pS308-DAPK1	Associated with ischemic neuronal cell death and brain infarction	[74]
		Increased DAPK1 protein level and enzymatic activity; Interaction with ERK	Promotes the ischemic reperfusion-induced neuronal apoptosis	[99]
		Binding to tau proteins in cortical neurons following brain ischemia	Phosphorylates the Ser262 of tau and causes dendritic spine injuries	[100]
		Association with the NMDAR subunit GluN2B at post-synaptic sites	Phosphorylates the Ser1303 of GluN2B and promotes intraneuronal Ca^2+^ overload and cell death	[73]
	Oxygen/glucose deprivation	Significant reduction of pS308-DAPK1; proteolytic cleavage of full-length DAPK1	Associates with ischemic neuronal cell death and brain infarction	[74]
		Interaction with p53 in primary neurons	Phosphorylates the Ser23 of p53 and triggers both cell apoptosis and necrosis	[101]
	Neonatal cerebral hypoxic ischemia by unilateral carotid ligation	DAPK1 catalytic activity elevated in the ischemic hippocampus in the late phase of hypoxic ischemia	May play a role in neuronal repair and differentiation in the recovery phase	[102]
	Two-vessel occlusion model (global ischemia)	Up-regulation of *DAPK1* mRNA in the cortex and hippocampus	Associated with ischemic neuronal cell death and brain infarction	[74]
Traumatic brain injury (TBI)	Diffuse axonal injury by lateral head rotation	Increased expression in hippocampal CA1 region and brainstem 1-day post injury, peaking at 3-day	Induces neuronal cell apoptosis and axonal degeneration	[103]
	Closed-head TBI by weight drop	Significant elevation of protein level at 48 h post-TBI in the cerebral cortex	Promotes abnormal tau phosphorylation and accumulation, thus leading to axonal injuries and cognitive impairments	[104]
	Controlled cortical impact of TBI	Elevated protein level at 1 week post TBI in the perilesional region of the cortex	Induces neuronal apoptosis and GluN2B phosphorylation	[105]
Epilepsy	Brief seizures induced by intra-amygdala kainic acid (KA) injection	Association with p53 and undergoes proteolytic cleavage in ipsilateral hippocampus	Mediates seizure-induced neuronal death in the hippocampus	[106]
	Pentylenetetrazol (PTZ) exposure-induced seizure	Robust increase in DAPK1 activity but not protein level in the cortex and hippocampus after acute PTZ treatment; remarkable upregulation of DAPK1 protein level in the cortex and hippocampus following chronic low-dose PTZ kindling	Stimulates the phosphorylation of GluN2B and induces seizure phenotypes	[76]
	Seizures induced by intraperitoneal injection of KA	Significant activation of DAPK1 revealed by an upregulation of ERK-induced pS735-DAPK1 in cortex and hippocampus after KA treatment	Triggers neuronal apoptosis and potentiates seizure activity	[77]
Parkinson’s disease (PD)	MPTP-induced mouse PD model	Remarkable increase of DAPK1 protein level in striatal neurons of MPTP-treated mice, while the mRNA expression is unchanged	Induces dopaminergic neuron death, promotes synucleinopathy and exacerbates motor deficits in mice	[82, 107, 108]
Depression	Chronic unpredictable stress (CUS)-induced rat depression model	Upregulation of both the protein level and the catalytic activity of DAPK1 in the medial prefrontal cortex of CUS rats; enhancement of DAPK1-GluN2B association	Disrupts the NMDAR signaling and induces synaptic dysfunction as well as depressive-like behavior	[109]
	CUS-induced mouse depression model	Significant increases in the protein level and activity of DAPK1 in the hippocampus of mice exposed to CUS	Facilitates abnormal tau phosphorylation and accumulation, and results in depressive-like behavior	[110]
Alzheimer’s disease (AD)	Brain tissues of AD patients	Marked increase in the protein level of DAPK1 in the hippocampus of AD patients compared with age-matched controls, while the mRNA expression levels are comparable	Associates with aberrant tau- and APP phosphorylation, and contributes to both tau and Aβ pathologies	[78, 79]
	Plasma samples of AD patients	Higher plasma DAPK1 protein level in AD than in control individuals	Negatively correlated with the cognitive ability	[111]
	Tg2576-APPswe mouse model	Age-dependent activation of DAPK1 in the excitatory pyramidal neurons in the entorhinal cortex of Tg2576 mice while the total DAPK1 protein level remains constant	Mediates synaptic degeneration in the excitatory pyramidal neurons of the entorhinal cortex	[112]
	Human tau (hTau) transgenic mouse model	Elevated DAPK1 protein level expression in cortex and hippocampus of 10-month hTau mice	Shows correlation with tau phosphorylation at Ser262	[113]
	PS1 V97L transgenic mouse model	Increased DAPK1 protein level in the hippocampus of 6-month and 9-month mice	Increases the Ser1303 phosphorylation of GluN2B	[111]
Huntington’s disease (HD)	YAC128 HD mouse model	Increased DAPK1 protein level and activation in the cortex and striatum of 1-month mouse HD model, while the DAPK1 mRNA level remains constant	Facilitates GluN2B phosphorylation at Ser1303 and damages synaptic functions	[114]

The dysregulation of DAPK1 is also noted in neurodegenerative proteinopathies such as AD, PD and TBI. For example, our previous research reported that the DAPK1 protein level but not its mRNA expression is significantly upregulated in the hippocampus of AD patients [78, 79]. Likewise, it has been shown that the 1-methyl-4-phenyl-1,2,3,6-tetrahydropyridine (MPTP)-induced PD mouse model displays a marked increase in DAPK1 protein level in the striatal neurons without showing alterations of mRNA expression. The specific DAPK1 alteration pattern in AD and PD might be caused by the post-transcriptional regulatory mechanisms such as microRNA (miRNA)-mediated translational control of the target protein. Several miRNAs targeting DAPK1 have been found to be dysregulated in AD or PD [80–82]. These findings highlight a detrimental role of DAPK1 dysregulation in the pathogenesis of age-related neurodegenerative diseases. The relationship between DAPK1 and AD will be elaborated in the following sections to clarify the genetic associations, functional changes and underlying mechanisms.

### Genetic association of DAPK1 with AD

Genetic variation is thought to play a major role in the etiology of LOAD, which accounts for about 95% of all AD cases. Genome-wide association studies (GWAS) have been used to identify single-nucleotide polymorphisms (SNPs) in genes associated with the risk and the age of onset of AD. A number of genetic loci contributing to the susceptibility to AD have been identified [83–87]. Among them, the ε4 allele of *ApoE* has been confirmed as the strongest genetic risk factor for AD in various populations [88, 89]. The risk of AD is increased by about 3 folds and 15 folds in individuals carrying one or two copies of *ApoE4* gene, respectively. The presence of *ApoE4* allele is also associated with a younger age of onset of AD symptoms [90].

Several genetic variants (e.g., the unc-13 homolog B gene) in chromosome 9 have been suggested to be connected with the risk of LOAD in whole-genome sequencing and parametric linkage analyses [91]. SNP analysis of *DAPK1*, which is also localized on chromosome 9, has identified two variants, rs4878104 and rs4877365, that are potentially connected with LOAD in several sample cohorts [36]. This discovery has sparked research on *DAPK1* SNPs and AD susceptibility in different populations, yielding controversial results regarding whether *DAPK1* SNPs are correlated with the risk of LOAD. Li et al. first reported that rs4878104 and rs4877365 of *DAPK1* influence the transcription of *DAPK1* in an allele-specific manner, although both SNPs are inside the *DAPK1* introns [36]. In particular, rs4878104 is located within a previously identified LOAD linkage peak and is repeatedly validated for its association with LOAD in six independent sample sets [36]. This finding was further corroborated by a genetic study in the Northern Han Chinese population, which showed that the genotype and the frequency of the rs4878104 but not the rs4877365 variant are significantly different between LOAD patients and age-matched controls [38]. Conversely, Minster et al. revealed that none of the *DAPK1* SNPs are associated with LOAD in their cohort [92]. Similar results were also reported by several GWAS analyses in French, Polish and Italian populations [37, 93, 94]. The inconsistent findings then led to systematic meta-analysis aiming at elucidating the association between *DAPK1* variants and AD, which discovered that the rs4878104 variant is not significantly associated with AD risk in the pooled population. Nevertheless, the subgroup analysis demonstrated that the rs4878104 variant is linked with AD risk in both American and Chinese populations but not in the European population [95]. Genotyping of rs4878104 revealed a discrepancy in the association between allele frequency and AD risk between Chinese and Caucasian populations, showing that the minor T allele is protective in Caucasians while the minor C allele is beneficial in Chinese population [36, 38].

In addition to the GWAS analysis, several biomarker-based studies have been performed to explore the association between DAPK1 and AD pathologies. For instance, Kauwe et al. reported that the minor allele of rs4878104 is associated with increased total CSF Aβ level [96]. However, by comparing brain samples from AD patients and healthy controls, Hainsworth et al. revealed that the expression of DAPK1 in the frontal cortex is not significantly different between AD and control cases. Moreover, the abundance of DAPK1 in the brain is also not correlated with Aβ level [97]. Thus far, there is still a lack of consistent molecular evidence to affirm the genetic association between *DAPK1* polymorphisms and the susceptibility to AD, likely due to the complex etiology of AD attributed to gene-environmental interactions [98]. Therefore, it is necessary to carry out large-scale genetic analysis under the latest Alzheimer’s diagnostic framework by applying genetic epidemiologic methods such as Mendelian randomization, to better clarify the exact association between *DAPK1* and AD phenotypes.

### DAPK1 dysregulation in AD patients and mouse models

The genetic link between *DAPK1* variants and AD has triggered further investigations on whether DAPK1 is dysregulated in the brains of AD patients or mouse models. In the adult brain, DAPK1 expression is relatively abundant in neurons of the hippocampus and cortex [39, 71], two brain regions that are significantly affected during AD progression. Hainsworth et al. first compared the protein level of DAPK1 in the frontal cerebral cortex between AD patients and age-matched controls. The overall abundance of DAPK1 evaluated by immunoblot and immunostaining analyses was not significantly different between AD and control samples, although the AD cases displayed a trend of increase in the immunoblot analysis [97]. To further determine whether hippocampal DAPK1 expression differs between AD and control brains, we analyzed hippocampal tissues from AD patients and age-matched controls, and discovered that the DAPK1 protein level was markedly increased by about 2.5 folds in the hippocampus of AD patients compared with that in normal subjects [78, 79]. However, there was no significant difference in DAPK1 mRNA expression between AD and control samples [79]. Notably, the upregulation of DAPK1 appears to correlate with tau hyperphosphorylation in our sample cohorts [79]. Since tau pathology occurs early in the entorhinal cortex and hippocampus during AD progression and may lead to irreversible synaptic dysfunction and neuronal loss [115], we hypothesize that the hippocampal DAPK1 dysregulation may also be an early pathological event in the disease course of AD. In line with this, a recent study discovered that the AD patients have significantly elevated plasma DAPK1 content compared with age-matched controls. In addition, this increase is negatively correlated with the cognitive ability [111]. Thus, the DAPK1 protein can be potentially developed as a clinical biomarker to monitor disease progression.

In AD mouse models, whether DAPK1 expression or function is dysregulated remains controversial in literature. Hainsworth et al. determined DAPK1 expression in brains of aged Tg2576 transgenic mice overexpressing the APP Swedish mutant and found that the DAPK1 protein level was comparable between AD mice and wild-type (WT) controls [97]. However, the expression of DAPK1 was significantly upregulated in the hippocampus of PS1 V97L transgenic mice compared with control mice at 6 and 9 months of age, which correlated with the appearance of Aβ accumulation and cognitive impairment [111]. Similarly, Duan et al. reported an elevation of DAPK1 expression in the hippocampus and cortex of 10-month-old human tau transgenic (hTau) mouse model, which paralleled the presence of hyperphosphorylated tau at Ser262 [113]. Nevertheless, none of the studies evaluated whether the enzymatic activity of DAPK1 is dysregulated in the brains of AD mouse models. Besides, whether DAPK1 is dysregulated in other AD mouse models of Aβ (e.g., APP/PS1 or 5×FAD) or tau (e.g., PS19 or tau P301L) pathologies remains to be characterized. Interestingly, it has been demonstrated that the expression of DAPK1 is elevated in the cortex overlying the corpus callosum in a mouse model of vascular dementia, which then drives abnormal accumulation of misfolded tau proteins in neurons and endothelial cells [116]. Brain ageing is tightly associated with the accumulation of cellular senescence in response to various stress signals, which may lead to structural and functional impairments in the central nervous system [117]. Guo et al. reported that the activity of DAPK1 is gradually increased whilst the protein level remains constant during the aging process in mouse hippocampus [118]. The age-dependent DAPK1 activation contributes to cognitive dysfunction in aged mice [118]. These findings indeed suggest that DAPK1 dysregulation plays a role in various cognitive disorders. Nevertheless, systematic analysis of DAPK1 expression and activity in the brains of AD mouse models is required to fully dissect the spatiotemporal association between DAPK1 and AD neuropathologies.

### Molecular mechanisms underlying DAPK1 dysregulation in AD

The protein level of DAPK1 is tightly controlled by both transcriptional and post-transcriptional mechanisms. Some transcriptional factors such as p53 and the cAMP response element-binding protein 1 (CREB1) have been shown to affect *DAPK1* transcription; however, their involvement in AD remains uncharacterized. Current evidence shows that the DAPK1 level is post-transcriptionally regulated in AD. In the following, we thus mainly focus on the post-transcriptional modulation of DAPK1 expression in AD.

Hyperactivation of p53 is an important molecular feature in several neurodegenerative diseases [119]. The p53 protein can bind to the first intron of *DAPK1* and efficiently reinforce the expression of DAPK1 in the presence of pro-apoptotic stimuli [120]. p53 upregulation in AD is mainly found in glial cells rather than in neurons [121]. Nevertheless, intracellular Aβ species also directly activate p53 expression by binding to its promoter [122]. Therefore, intracellular Aβ accumulation might upregulate DAPK1 expression through p53-mediated pathways.

CREB1 is a well-known transcription factor in neurons. CREB1 is enriched on the promoter of *DAPK1* and directly suppresses its transcription in the hippocampus. Reduced neuronal CREB expression may result in elevated DAPK1 expression and subsequent cell death, as has been reported in a rat brain injury model induced by cardiac arrest [123]. The CREB signaling is impaired in the brains of AD patients, as manifested by a significant decline in both total and activated CREB levels in AD subjects compared with those in normal controls [124]. Thus, the CREB-mediated transcriptional control of *DAPK1* might be an alternative pathway that modulates DAPK1 expression in AD. Contrary to these findings, our previous studies established that the upregulation of DAPK1 expression in AD is not caused by transcriptional changes [78, 79]. These results suggest that the expression of DAPK1 in AD is likely regulated through both transcriptional processes and post-transcriptional pathways that are similar to those of the glycogen synthase kinase-3β (GSK-3β) and CDK5 [125, 126].

**Fig. 2:**
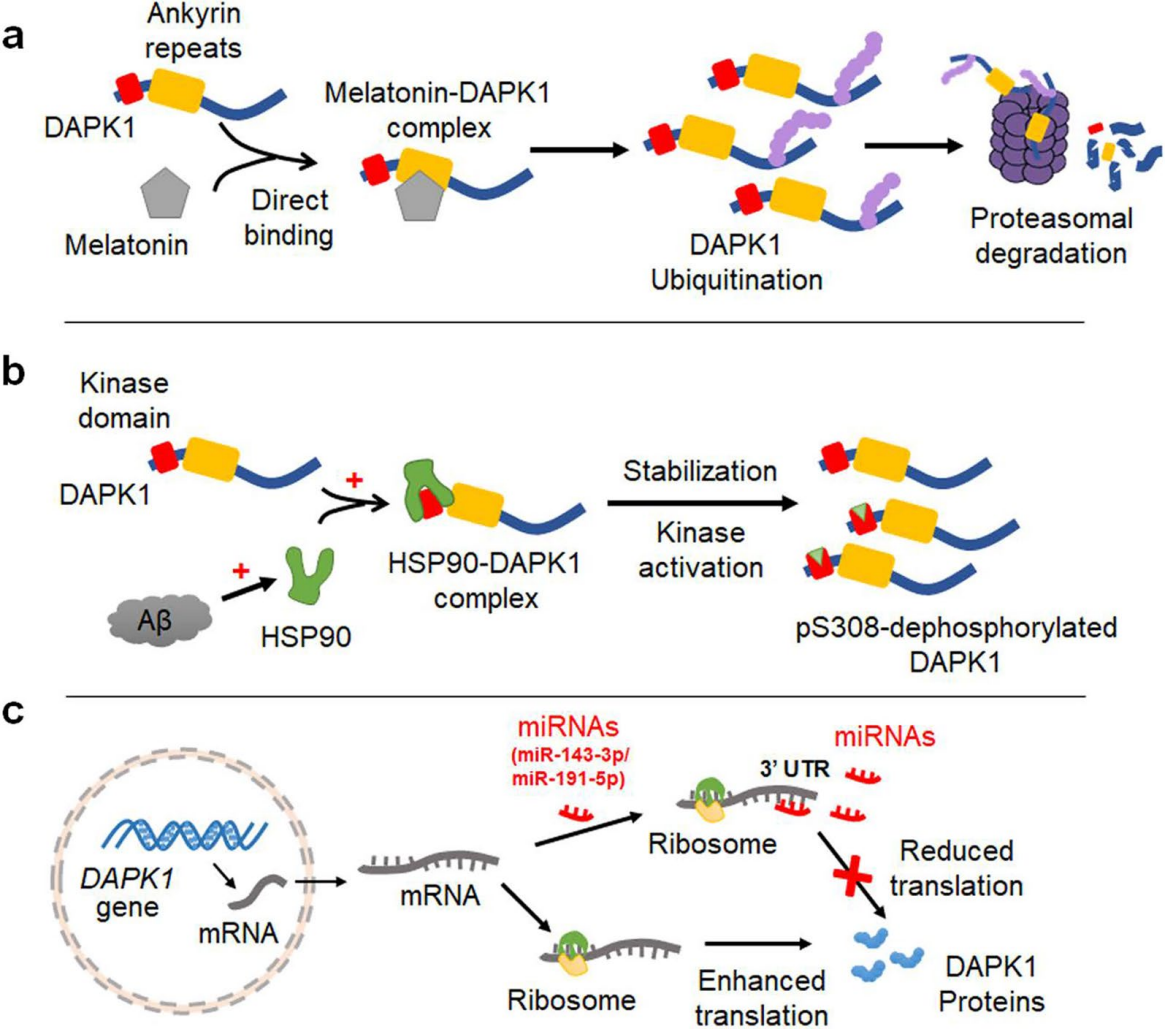
Potential molecular mechanisms underlying DAPK1 dysregulation in AD. **a** Melatonin directly binds to the ankyrin repeats of DAPK1, promoting its ubiquitination and subsequent degradation in proteasomes. In AD, a downregulation of melatonin in the brain causes a reduction of proteasomal-degradation of DAPK1, leading to increased DAPK1 protein levels in the brain. **b** HSP90 is activated by Aβ accumulation in neurons. The activated HSP90 interacts with the kinase domain of DAPK1, resulting in the stabilization and activation of DAPK1 in the brain. **c** Some miRNAs (such as miR-143-3p and miR-191-5p) directly target the 3’ UTR of DAPK1 mRNA, leading to a translational repression of DAPK1 expression. In AD, the downregulation of these miRNAs may significantly promote the translation of DAPK1 mRNA, thereby elevating DAPK1 protein contents in the brain

Circadian dysregulation is tightly associated with the progression of AD [127]. Melatonin is a master regulator of human circadian rhythm and confers numerous beneficial effects on neuronal function [128]. The brain level of melatonin is lower in AD patients than in normal controls [129]. Since melatonin is capable of preventing tau hyperphosphorylation and Aβ accumulation in AD mouse models, its downregulation may increase the vulnerability to AD [130, 131]. We recently uncovered an inverse correlation between brain melatonin level and DAPK1 expression in AD patients [129]. Furthermore, melatonin directly binds to the ankyrin repeats of DAPK1, leading to the ubiquitination and proteasome-dependent degradation of DAPK1 in cells (Fig. 2a) [129]. Our study also proved that the melatonin-induced DAPK1 downregulation does not rely on melatonin receptors or *DAPK1* gene transcription [129]. Indeed, the post-translational regulation of DAPK1 level in the cell is primarily controlled by the proteasomal and lysosomal pathways [132]. Several binding partners of DAPK1, including HSP90 and DIP-1, have been shown to regulate the stability of DAPK1 protein [47]. Molecular chaperones are key components for the maintenance of proteostasis in the cell by monitoring the proper folding, maturation and degradation of proteins [133]. With accumulation of intracellular Aβ species, molecular chaperones such as HSP90 may function to antagonize the cellular stress caused by the excessive misfolded proteins [134]. We recently observed that the primary neurons incubated with Aβ aggregates have a higher DAPK1 protein level than those without Aβ treatment [135]. However, the DAPK1 mRNA level is not altered by Aβ, which is in line with our previous findings in brain samples from AD patients [79]. The increased DAPK1 protein level by Aβ treatment is attributed to the HSP90-mediated stabilization of DAPK1 protein in neurons, as inhibition of HSP90 abolishes the Aβ-induced DAPK1 elevation (Fig. 2b) [135].

In addition to the protein stability regulation, DAPK1 expression may also be affected by various miRNAs. Our group has identified two miRNAs, miR-143-3p and miR-191-5p, as important upstream regulators of DAPK1 expression in AD. Levels of both miRNAs are significantly reduced in brains of AD patients, and are negatively correlated with DAPK1 protein level [80, 81]. Moreover, miR-143-3p and miR-191-5p bind to the 3′ UTR of *DAPK1* mRNA. Instead of inducing mRNA degradation, they block the translation of cellular DAPK1 (Fig. 2c) [80, 81]. Several other miRNAs have been reported to regulate DAPK1 expression in the brain. For instance, *DAPK1* is a target gene of miR-130a-3p. The downregulation of miR-130a-3p induced by Aβ treatment promotes DAPK1 expression, resulting in accelerated neuronal cell death and cognitive impairment [136]. MiR-124 binds to the 3′ UTR of *DAPK1* mRNA and affects the translation of DAPK1 in ischemic stroke models [137]. In PD, a loss of miR-26a leads to an elevation of DAPK1 protein level in dopaminergic neurons [82, 138]. Interestingly, it has been reported that the level of miR-26a is significantly decreased in AD patients and mouse models [139, 140], and is closely associated with the cognitive ability in AD patients [139]. Therefore, it is expected that miR-26a dysregulation may also contribute to the DAPK1 upregulation in AD.

## The role of DAPK1 in Alzheimer’s core pathologies

### The role of DAPK1 in tau pathology

**Fig. 3:**
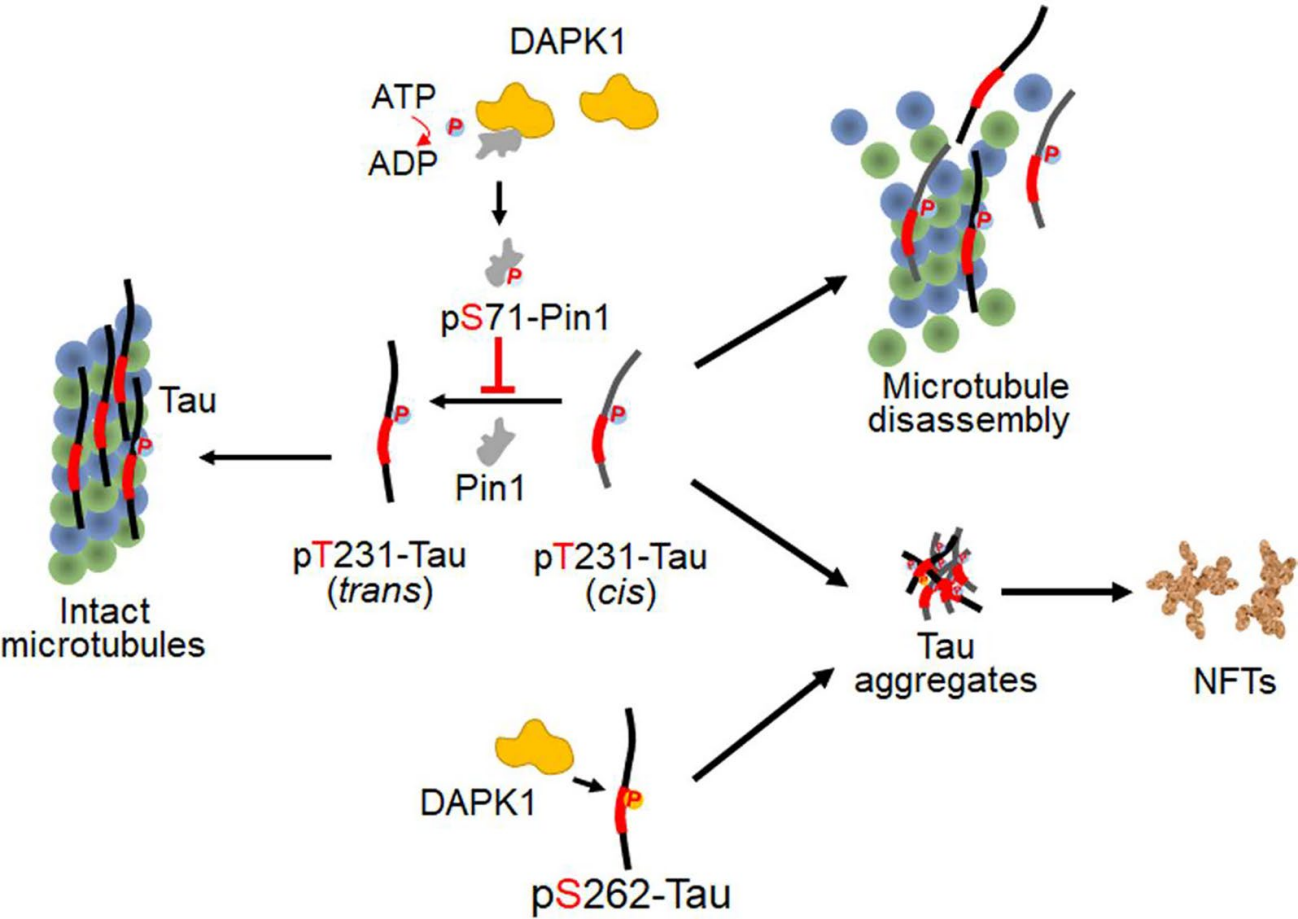
DAPK1 dysregulation induces tau hyperphosphorylation and microtubule injury in AD. Neuronal tau is essential for regulating microtubule dynamics. DAPK1 dysregulation contributes to tau pathology through two mechanisms. First, DAPK1 interacts with Pin1 and suppresses its activity by directly phosphorylating the Ser71 residue of Pin1. Upon phosphorylation, the Pin1-mediated *cis*-to-*trans* isomerization of pThr231-Pro motif in tau protein is decelerated, leading to the accumulation of *cis* pT231-tau that is resistant to degradation/dephosphorylation and has lower microtubule binding affinity but higher aggregation propensity. Second, it has been reported that DAPK1 directly interacts with tau and phosphorylates its Ser262 residue, thereby exacerbating neuronal tau pathologies

The abnormal phosphorylation and accumulation of tau in the brain is closely associated with the disease course of AD, and has been used as a clinical biomarker for disease diagnosis [141]. Tau has about 85 putative phosphorylation sites, and around 50 sites, mostly located in the basic C-terminal half of the protein, have been identified in brain tissue [141, 142]. Phosphorylation modulates the surface charge of tau and its conformation, thus altering the inter- and intra-molecular interactions important for the physiological functions of tau in neurons [143]. In pathological conditions known as tauopathies, neuronal tau proteins are hyperphosphorylated at multiple sites, leading to the detachment of tau from microtubules and subsequent misfolding and aggregation, which ultimately drives microtubule disassembly and the formation of pathogenic tau aggregates [7]. It has been noted that the aberrant tau phosphorylation in AD is not only characterized by a 3- to 4-fold increase in overall phosphorylation level, but also by the presence of various novel modified sites not seen in healthy brains [144]. DAPK1 has been found to be upregulated in brains of hTau transgenic mouse models. By overexpressing or knocking down DAPK1 in HEK 293 cells stably expressing tau proteins, Duan et al. found that DAPK1 increases tau phosphorylation at Thr231, Ser262 and Ser396 sites [113]. Interestingly, DAPK1 has no effect on common tau phosphorylating kinases such as GSK-3β, CDK5, CaMKII and Cdc2 or protein phosphatase 2A (PP2A). However, DAPK1 overexpression elevates the activity of microtubule affinity-regulating kinase 2 (MARK2) through direct binding between the death domain of DAPK1 and the spacer region of MARK2 [113, 145]. MARK2 is known to interact with and phosphorylate tau at Ser262 residue [146]. MARK2 knockdown has no impact on DAPK1-induced tau hyperphosphorylation [113], but the MARK2-induced tau phosphorylation and microtubule destabilization are potentiated by DAPK1 in neurons as DAPK1 overexpression enhances MARK2 activity in tauopathy [145]. Interestingly, DAPK1 is able to interact with the tau protein through its kinase domain, further leading to tau phosphorylation at Ser262 [100], promoting dendritic spine damage and neuronal death in ischemic stroke mice [100]. Our group further identified a novel mechanism by which DAPK1 increases neuronal tau hyperphosphorylation and accumulation in AD. We discovered that DAPK1 aggravates neuronal tau phosphorylation at Thr231, Ser262 and Ser396 by inhibiting the Pin1-mediated *cis–trans* isomerization of the pThr231-Pro motif, which is required to maintain normal tau functions and turnover (Fig. 3) [79, 147, 148]. This unique mechanism was further validated by the observation that DAPK1 enhances the protein stability of S262A (Ser to Ala) tau mutant, while having no effect on the T231A (Thr to Ala) mutant [79]. The DAPK1-induced tau hyperphosphorylation promotes the formation of sarkosyl-insoluble tau species (mature pathogenic NFTs), disintegrates microtubule structures and prevents neurite outgrowth [79]. Besides, the accumulation of *cis*-pThr231-tau caused by DAPK1 upregulation robustly triggers axonal damage and neuroinflammation in TBI mouse model [104]. Our recent study also demonstrated that toxic Aβ species facilitate tau hyperphosphorylation and accumulation in neurons by upregulating DAPK1 expression [135]. Inhibition of DAPK1 activity with synthetic small molecules antagonizes pathological tau phosphorylation and accumulation caused by TBI or Aβ species [104, 135].

As a pro-apoptotic kinase, the upregulation of DAPK1 is believed to stimulate cell apoptosis under stress stimuli. However, it has been shown that tau hyperphosphorylation following DAPK1 activation exerts anti-apoptotic effects in N2a cells by blocking the generation of cleaved caspase-3 [113]. Previous research has reported that tau accumulation and hyperphosphorylation protect cells from apoptosis via reducing the p53 level and preserving mitochondrial functions [149]. DAPK1 can bind to p53 via the death domain and phosphorylate the Ser23 residue of p53, further triggering the p53-dependent transcription of apoptotic genes and mitochondrial damage in ischemic stroke [101, 150]. The Aβ species-induced DAPK1 upregulation in primary neurons leads to both tau hyperphosphorylation and caspase-3-mediated apoptosis [135]. Thus, it remains debatable as to whether DAPK1-induced tau phosphorylation confers anti-apoptotic effects in neurons. In addition to AD and TBI, it has been reported that chronic stress exposure in mice concomitantly elevates the expression of DAPK1 and abnormal tau phosphorylation in the hippocampus, which can be reversed by exercise training and antidepressant treatment [110]. These results together suggest that DAPK1 dysregulation indeed causes tau hyperphosphorylation and accumulation, and promotes neuronal damage by disrupting the microtubule dynamics in AD and other neurological disorders.

### The role of DAPK1 in APP processing, Aβ secretion, and Aβ-induced neurotoxicity

**Fig. 4:**
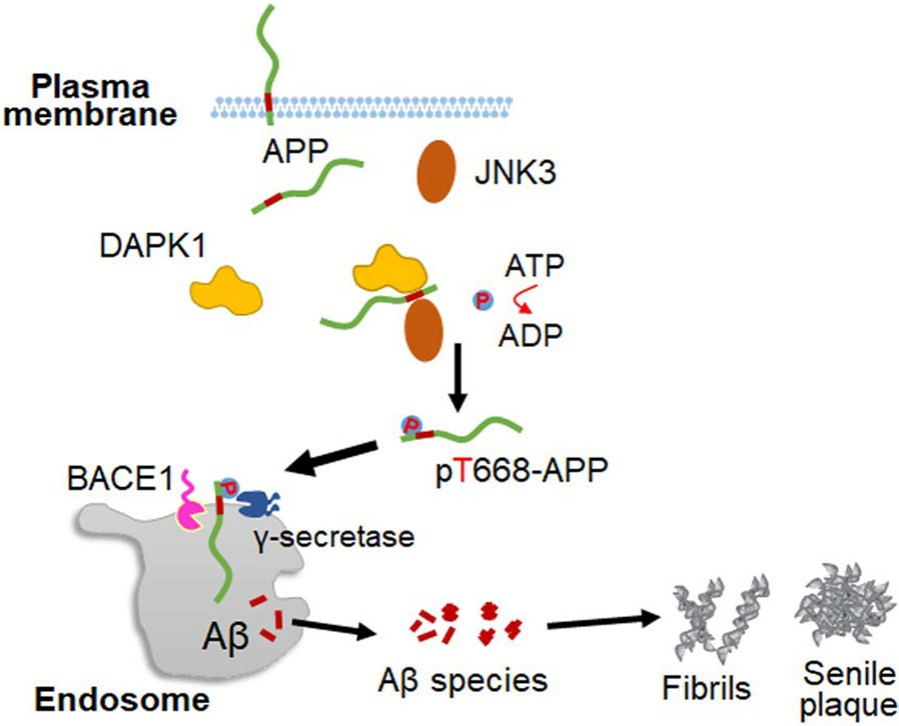
DAPK1 dysregulation triggers APP phosphorylation and enhances the amyloidogenic processing pathway. DAPK1 is able to interact with APP in neurons, and potentiates the JNK3-induced APP phosphorylation at the Thr668 residue. Phosphorylated APP translocates to the endosomes and undergoes the amyloidogenic cleavage by BACE1 and γ-secretases sequentially. The generation of Aβ is thus enhanced, leading to the pronounced formation of amyloid plaques in the brain

The accumulation and aggregation of Aβ in the brain parenchyma is an early pathological change in AD, occurring decades prior to the development of clinical symptoms [151]. The generation of Aβ by the amyloidogenic processing of APP is affected by the post-translational modification of APP in neurons [152]. Notably, the phosphorylation of APP in the intracellular domain modulates multiple steps of the proteolytic cleavage process and has a profound influence on the production of Aβ [33]. Eight phosphorylation sites have been identified in the cytoplasmic domain of APP thus far, and seven of them have been discovered in the brains of AD patients [33]. The Thr668 residue of APP (APP695 numbering) can be phosphorylated by a variety of kinases related to cell proliferation and stress responses [153]. Moreover, Thr668 phosphorylation has been proven to be highly upregulated in the brains of AD patients compared with age-matched controls. The phosphorylation of Thr668 facilitates the translocation of APP to endosomes, thus promoting the colocalization of APP with the β-secretase BACE1 [32]. As a consequence, the generation of Aβ is dramatically increased in neurons due to the enhanced amyloidogenic processing. Recently, we showed that DAPK1 interacts with APP through its death domain and increases the JNK3-mediated APP phosphorylation at Thr668, thereby shifting APP processing toward the amyloidogenic pathway (Fig. 4) [78]. DAPK1 strongly promotes the secretion of both Aβ40 and Aβ42, and ablation of DAPK1 significantly diminishes the production of Aβ in Tg2576 mouse model [78]. Besides, we observed a positive correlation between DAPK1 protein levels and APP phosphorylation at Thr668 in Alzheimer’s patient brains, further supporting the role of DAPK1 in regulating the amyloid pathology in AD [78]. We additionally revealed that pharmacological inhibition of DAPK1 activity by a selective DAPK1 inhibitor ((4Z)-4-(3-pyridylmethylene)-2-styryl-oxazol-5-one) significantly downregulates the amyloidogenic processing of APP and Aβ generation in different cell models, suggesting that DAPK1 is a promising target for early intervention of Aβ pathology in AD.

Similar to the pThr231-Pro motif of tau, the pThr668-Pro motif of APP is also a substrate of Pin1-mediated *cis–trans* isomerization. The phosphorylation of Thr668 is essential for the binding between Pin1 and APP at the plasma membrane [154]. Pastorino et al. found that Pin1 potently catalyzes the conversion of the *cis* pThr668-Pro APP conformers to the *trans* conformation which is preferentially favored by the non-amyloidogenic processing in neurons [154]. Since DAPK1 is known to phosphorylate Ser71 of Pin1 and inhibits the prolyl isomerase activity [57], we speculate that the DAPK1-induced APP phosphorylation and the *cis* pThr668-Pro APP accumulation caused by DAPK1-induced Pin1 suppression both contribute to the excessive generation of Aβ in AD.

In addition to affecting APP processing and Aβ generation, DAPK1 dysregulation is also involved in mediating Aβ neurotoxicity in the brain. The self-assembly of Aβ into soluble oligomers has been recognized as a main culprit for Aβ-related neuropathological changes [155]. Low-molecular-weight Aβ species activate DAPK1 by inducing the dephosphorylation of Ser308 and trigger caspase-3-dependent neuronal cell death [156]. We recently demonstrated that both low- and high-molecular-weight Aβ species upregulate DAPK1 protein levels in neurons and cause extensive neuronal apoptosis [135]. Additionally, the upregulation of DAPK1 by Aβ oligomers leads to Ca^2+^ overload and excitotoxicity in the SH-SY5Y cell line via phosphorylation of the Ser1303 residue of the NMDAR GluN2B subunit, resembling the effect observed in an ischemic stroke mouse model [73, 111]. Neuroinflammation is another prominent pathological change in AD, characterized by the presence of gliosis and abundant pro-inflammatory cytokines in cortex and hippocampus. Song et al. found that the Aβ species stimulate DAPK1 activity in BV2 microglial cell line via activating the lysosomal cathepsin B [157], thereby promoting the generation of interleukin-1β (IL-1β) and subsequent cognitive impairments in vitro and in vivo [157].

### DAPK1 and neuronal cell death in AD

Neuronal cell death is commonly observed during neural development and pathological conditions [158]. The programmed cell death in the developmental period contributes to the elimination of redundant neurons, which is crucial for the establishment of functional neural circuits and the maintenance of normal organ size [158]. The developmental cell death occurs in a spatiotemporally restricted and well-controlled manner to regulate the functional and structural homeostasis in the brain [159]. However, abnormal cell death in specific neuronal populations is a pathological hallmark of neurodegenerative diseases including AD, and is closely associated with functional and structural impairments during disease progression [3]. For example, cholinergic neurons in the basal forebrain are a primary cluster of neurons forming neural connections with the cortex, hippocampus and amygdala [160]. They regulate a wide range of physiological functions such as learning and memory, attention and emotion [161]. In pathological conditions such as AD, cholinergic neurons undergo irreversible degeneration and cell death, ultimately leading to brain atrophy and cognitive decline [162]. The specific spatial and temporal expression of DAPK1 in the brain implicates a potential role of DAPK1 in regulating neuronal cell apoptosis during neural development [39, 163], yet systematic research is needed to fully reveal the exact role of DAPK1 in early brain development. Surprisingly, we and others noticed that whole-body *DAPK1* knockout (KO) in mouse has minimal influence on the overall lifespan and brain morphology [164]. Besides, DAPK1 KO or kinase activity deficiency in mice does not cause behavioral deficits [104, 165]. These findings indicate that the physiological role of DAPK1 in neural development might be compensated by other mechanisms in KO mouse models, but this needs further evidence.

**Fig. 5:**
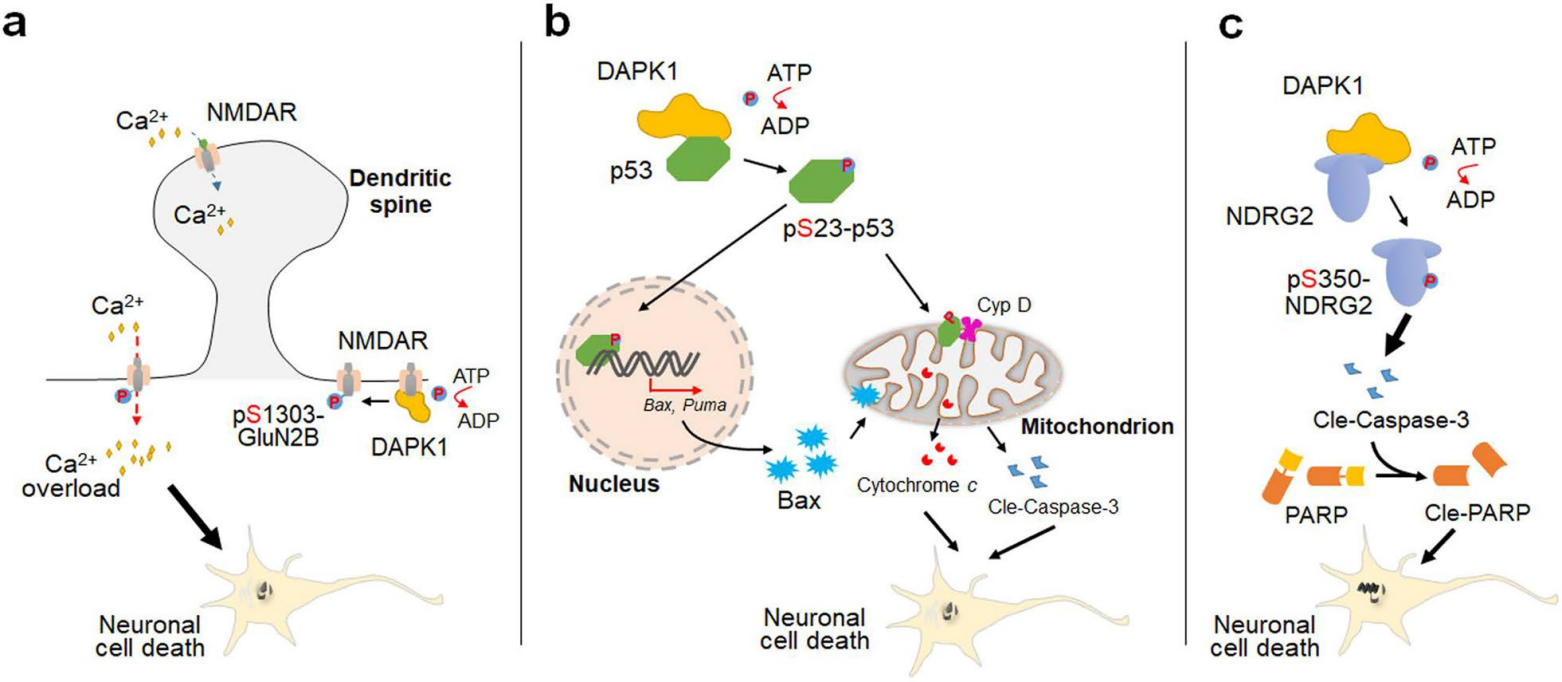
DAPK1 dysregulation leads to neuronal cell death through multiple pathways. **a** DAPK1 interacts with the GluN2B subunit of extrasynaptic NMDAR and phosphorylates the Ser1303 of GluN2B, thereby elevating the channel conductance of NMDAR toward Ca^2+^. Ultimately, neurons experience extensive excitotoxicity and undergo apoptosis due to Ca^2+^ overload. **b** DAPK1 directly phosphorylates the Ser23 of p53. Phosphorylated p53 enters cell nucleus to promote the transcription of pro-apoptotic genes such as *Bax* and *Puma*, leading to neuronal cell death in ischemic stroke. Besides, it can anchor to mitochondrial membranes and disrupt membrane integrity by interacting with the cyclophilin D (Cyp D), causing the release of cytochrome *c* and the generation of cle-caspase-3. **c** DAPK1 upregulation causes the phosphorylation of the Ser350 residue of NDRG2, resulting in the activation of caspase-3-dependent neuronal cell death

The dysregulation of DAPK1 in neuronal cell death was first demonstrated in ischemic stroke. Activated DAPK1 phosphorylates the Ser1303 residue of GluN2B at extrasynaptic sites, leading to enhanced NMDAR channel conductance and subsequent Ca^2+^ influx in neurons (Fig. 5a) [73]. Blocking DAPK1 rescues the Ca^2+^ overload-induced ischemic neuronal death and brain damage [73]. The excitotoxicity of GluN2B phosphorylation elicited by DAPK1 activation is also involved in neuronal loss caused by epilepsy, toxic Aβ treatment, stress-related depression and TBI insult [76, 77, 105, 109]. An earlier study demonstrated that DAPK1 binds to and phosphorylates p53 at the Ser23 residue. Upon the Ser23 phosphorylation, p53 translocates to the nucleus to initiate pro-apoptotic gene transcription. In addition, phosphorylated p53 also interacts with cyclophilin D in mitochondria and evokes mitochondrion-dependent necrosis (Fig. 5b) [101]. Previous studies have shown that DAPK1 can trigger both cell apoptosis and autophagic cell death [166]. A notable feature of DAPK1-induced cell death is cellular morphological changes such as membrane blebbing and cell protrusion, which are attributed to the phosphorylation of myosin-II light chain at Ser19 by DAPK1 and the subsequent alterations in cytoskeletal structure [166, 167]. We also identified N-myc downstream-regulated gene 2 (NDRG2) as a novel DAPK1 substrate in regulating neuronal cell death [168]. DAPK1 interacts with NDRG2 via its cytoskeleton-binding region, and directly phosphorylates the Ser350 of NDRG2 [168]. DAPK1-induced NDRG2 phosphorylation mediates the neuronal cell apoptosis triggered by Aβ species or ceramide through activating the caspase-3 pathway (Fig. 5c) [168]. Furthermore, NDRG2 phosphorylation at Ser350 is significantly elevated in the Tg2576 mouse model and in hippocampal tissues of AD patients. *DAPK1* KO successfully reverses hippocampal neuronal loss in 8-month-old Tg2576 mice [168]. These findings support a causal role of DAPK1 dysregulation in AD-related neuronal cell death.

Dysregulation of sphingomyelin has been suggested to be associated with the progression of cognitive decline and hippocampal atrophy in AD [169]. Ceramide is a metabolite from enzymatic cleavage of sphingomyelin in neurons and regulates a broad range of cellular events including senescence, survival, and apoptosis [170]. Brain regions with degenerative phenotypes show elevated ceramide content. The accumulation of ceramide has been shown to activate DAPK1 by inducing PP2A-mediated dephosphorylation of the Ser308 residue, thereby causing hippocampal neuronal death [171, 172]. In addition to sphingomyelin dyshomeostasis, the missense mutation (T835M) of the netrin receptor uncoordinated-5 homologue C (*UNC5C*) contributes to the risk of LOAD by increasing the vulnerability of neurons to cell death without affecting Aβ pathologies [173]. Hashimoto et al. demonstrated that the T835M-UNC5C interacts with DAPK1 via the respective death domain and induces pronounced cell death by activating the JNK/NADPH oxidase/caspase pathway [174]. Pharmacological inhibition of DAPK1 activity attenuates the T835M-UNC5C-induced cell death, suggesting that the interaction between UNC5C and DAPK1 may upregulate the catalytic activity of DAPK1 [174]. Indeed, it has been revealed that the Unc-5 homolog 2, a member of the UNC5 family, interacts with DAPK1 and recruits a subunit of PP2A to dephosphorylate the Ser308 residue of DAPK1, leading to DAPK1 activation and extensive cell death [175, 176]. Thus, these results all corroborate the fundamental role of DAPK1 in modulating neuronal cell death in the pathogenesis of AD.

### The role of DAPK1 in synaptic functions in AD

**Fig. 6:**
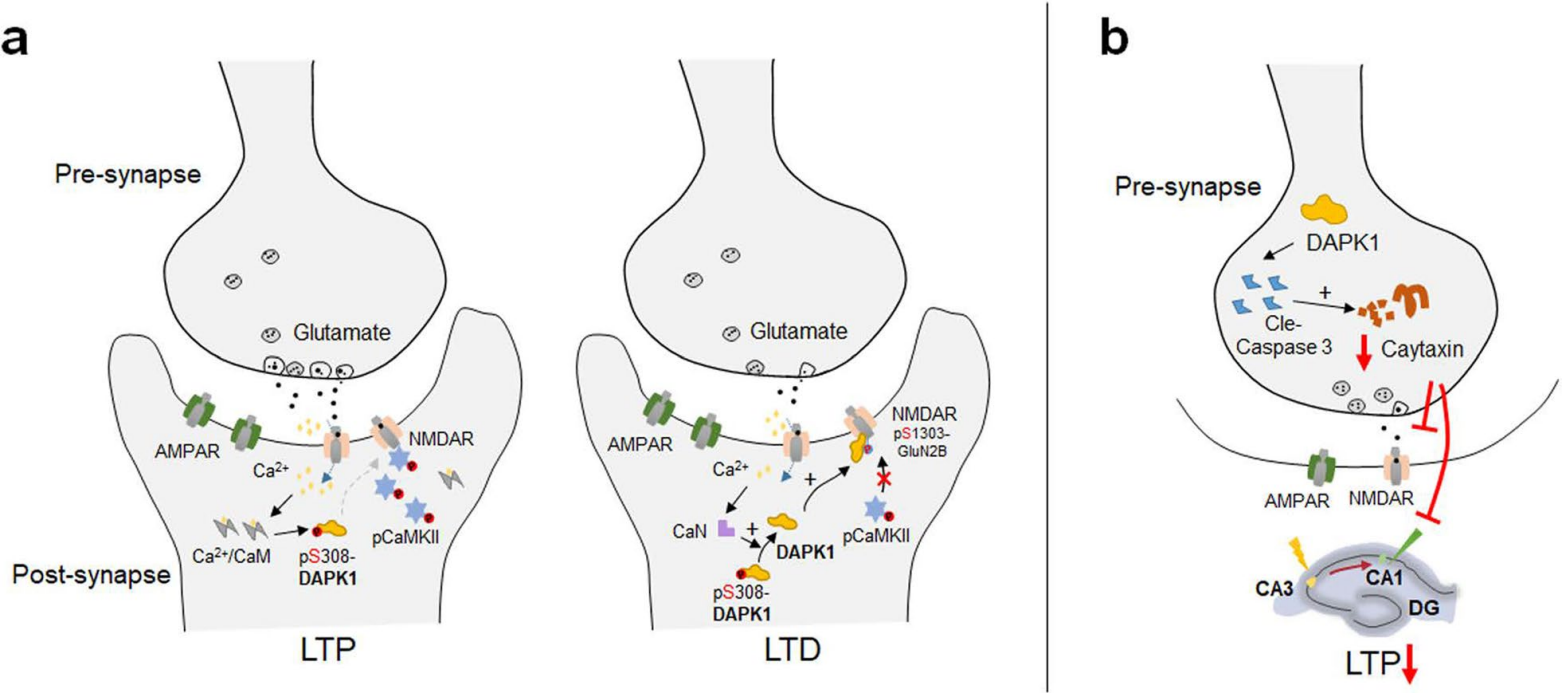
The role of DAPK1 in regulating synaptic functions. **a** DAPK1 is enriched in neuronal synapses, and is physiologically involved in the modulation of synaptic plasticity by interacting with the GluN2B subunit of NMDAR. During LTP, high levels of Ca^2+^/CaM suppress the DAPK1-induced phosphorylation of GluN2B, which then facilitates the binding between CaMKII and NMDAR and potentiates LTP formation. However, in LTD, DAPK1 is activated by calcineurin-mediated dephosphorylation of Ser308 residue, leading to an upregulation of GluN2B phosphorylation at Ser1303 that is necessary for the formation of LTD. **b** It has also been reported that DAPK1 is hyperactivated in the hippocampus during brain ageing. The activation of DAPK1 results in caspase-3-dependent Caytaxin degradation in presynapses, further affecting synaptic neurotransmission and the formation of LTP

Synapses are the structural unit for neuronal communications and form the functional basis for neural circuits that are vital for brain function. The progressive degeneration of synapses in the brain is highly correlated with cognitive decline in AD [177], and is an emerging therapeutic target for disease-modifying treatment of AD. The high abundance of DAPK1 in adult hippocampal tissues and in particular its enrichment in dendritic spines indicate that DAPK1 may possess important physiological functions in regulating synaptic structure and plasticity [73, 164]. This has been confirmed by our recent quantitative proteomic and phosphoproteomic analyses of hippocampal tissues from WT and *DAPK1* KO mice [178]. Glutamate receptors are one of the major modulators of the synaptic Ca^2+^ influx in neurons [179]. NMDARs dynamically change their subcellular localization, trafficking and expression levels in reaction to neuronal activities, and are believed to be critical for long-term potentiation (LTP) and long-term depression (LTD) [180]. Importantly, the DAPK1-induced Ser1303 phosphorylation of the NMDAR GluN2B subunit, which was originally reported to be detrimental in ischemic stroke, has essential physiological functions in modulating LTP and LTD (Fig. 6a) [181]. It has been revealed that DAPK1 and CaMKII differentially bind to GluN2B in LTD and LTP, respectively. LTP stimuli induce high levels of cellular Ca^2+^/CaM that can disrupt the binding between DAPK1 and GluN2B while facilitating the interaction between CaMKII and GluN2B. Besides, DAPK1 is dispersed from dendritic spines during LTP due to the transient depolymerization of F-actin [72, 181]. However, LTD stimuli trigger the calcineurin-mediated DAPK1 activation by dephosphorylating the Ser308 residue. This then causes an increase in the DAPK1–GluN2B interaction and subsequent phosphorylation of the Ser1303 residue, which retains DAPK1 in dendritic spines but prevents the access of CaMKII to GluN2B [72, 181]. It should be mentioned that the transient activation of DAPK1 is required for LTD induction under physiological conditions, which differs from the sustained upregulation of DAPK1 in ischemic stroke or AD [181]. Excessive DAPK1 in the synapse may prohibit the synaptic accumulation of CaMKII and result in LTP deficits in the brain. Aβ aggregates induce prolonged upregulation of DAPK1 in primary neurons [135], which may further mediate the NMDAR overactivation-induced Ca^2+^ dyshomeostasis and excitotoxicity in AD brains. Shu et al. demonstrated that DAPK1 is selectively activated in excitatory pyramidal neurons of the entorhinal cortical layer II region (ECII_*PN*_) prior to the appearance of Aβ deposition [112]. The activation of DAPK1 in this brain region is strongly involved in synaptic dysfunction manifested by significantly reduced presynaptic terminals and dendritic spines, and disordered excitatory and inhibitory balance in CA1 region [112]. Inhibition of DAPK1 activity in ECII_*PN*_ potently improves the synaptic transmission and cognitive performance of AD mice [112]. Therefore, DAPK1 may contribute to synaptic degeneration during AD progression irrespective of the presence of Aβ species through different molecular mechanisms.

DAPK1 has also been shown to interact with several presynaptic proteins including Caytaxin, Syntaxin-1A and α-synuclein (α-syn) [71, 108, 182]. For example, the upregulation of DAPK1 during brain ageing is associated with a downregulation of presynaptic Caytaxin levels, which is likely mediated by the DAPK1-induced caspase-3 activation [118]. A loss of Caytaxin expression significantly impairs the frequency and the amplitude of neuronal miniature excitatory postsynaptic currents and weakens hippocampal LTP in vivo (Fig. 6b) [118]. Interestingly, the interaction between DAPK1 and Caytaxin also reciprocally inhibits the presynaptic DAPK1 activity and protects neurons against the ischemic stroke-induced apoptosis [182], representing an early protective mechanism by which neurons antagonize ischemic brain injuries. Syntaxin-1A is a basic component in the regulation of neurotransmitter release. It participates in the synaptic vesicle docking and fusion process by cooperating with other proteins in the SNARE complex [183, 184]. Tian et al. found that DAPK1 associates with the C-terminal half of Syntaxin-1A in the plasma membrane, and phosphorylates its Ser188 residue in a Ca^2+^-dependent manner. The phosphorylated Syntaxin 1A has reduced binding affinity to Munc18, while the assembly of the SNARE complex is not affected [71].

α-Syn is another binding partner of synaptic vesicles in neurons and is closely associated with the pathogenesis of PD. DAPK1 phosphorylates α-syn at the Ser129 residue, promoting the self-aggregation of α-syn into toxic species both in vitro and in vivo [82, 108]. However, it is not clear how the DAPK1-induced α-syn alteration modulates synaptic functions. Goodell et al. revealed that *DAPK1* KO mice have comparable LTP strength to that of WT mice, while the LTD level is enhanced at a young age [164]. Functional studies demonstrated that *DAPK1* KO mice have different presynaptic components in hippocampus compared with the WT mice, as evidenced by the increased paired-pulse ratio and reduced glutamate release probability in the hippocampus of KO mice [164]. Our transcriptomic analysis in *DAPK1* KO and WT mice suggested that DAPK1 ablation globally changes the transcriptional profile in multiple brain regions and significantly regulates genes related to glutamatergic and GABAergic synaptic pathways [185]. The potential involvement of DAPK1 in modulating synaptic transmission and plasticity hints that DAPK1 dysregulation in AD may additionally cause synaptic impairments. However, in vivo studies using both *DAPK1* KO and transgenic AD mouse models are needed to fully address how DAPK1 is involved in synaptic deficits during AD progression.

### DAPK1 and other AD pathological changes

The progression of AD is also accompanied by gliosis, chronic neuroinflammation, oxidative stress and autophagy deficiency [186]. The sustained activation of glial cells in the brain not only leads to an imbalance in pro- and anti-inflammatory signals, but also impairs the clearance of debris and protein aggregates, and disrupts synaptic integrity [187, 188]. Therefore, the management of gliosis represents a promising approach to counteracting neuroinflammatory response in AD [189]. Previous research indicated that DAPK1 expression is rather low or undetectable in glial cells at the embryonic stage [39]. However, the expression pattern of DAPK1 in glial cells in the adult brain or in response to external stimuli remains uncharacterized. Song et al. found that DAPK1 is expressed in the BV2 murine microglial cell line. The activity of DAPK1 but not its protein level can be upregulated by Aβ treatment in lipopolysaccharide-primed BV2 cells [157]. The activation of DAPK1 promotes the production of IL-1β and the initiation of caspase 1-mediated activation of the NOD-, LRR- and pyrin domain-containing 3 (NLRP3) inflammasome, further leading to memory deficits in mouse models [157]. The Aβ treatment-induced activation of DAPK1 may be attributed to the elevation of cytosolic cathepsin B, which is able to interact with DAPK1 and slow down the turnover of endogenous DAPK1 [157, 190]. Pharmacological inhibition of DAPK1 activity attenuates the Aβ-induced NLRP3 activation and cognitive impairment in vivo. These findings implicate that modulating DAPK1 function in microglia might have protective effects on AD-related chronic neuroinflammation.

In addition to regulating the inflammatory response, microglia are also actively involved in maintaining brain homeostasis by executing phagocytosis of redundant synapses, damaged cells, myelin debris and protein aggregates [191]. It has been demonstrated that the autophagy-lysosomal system, an essential cellular machinery for the phagocytosis process, is impaired in the microglia of AD mouse models [192–194]. DAPK1 is an upstream regulator of cellular autophagy that directly interacts with proteins involved in the autophagy machinery. For instance, DAPK1 phosphorylates the Thr119 of Beclin1, and thus facilitates the release of Beclin1 from Bcl-X_L_ to initiate the autophagy process [195, 196]. Besides, it has been reported that DAPK1 forms a stable complex with MAP1B in cells subjected to starvation. MAP1B is indispensable for DAPK1-induced autophagosome accumulation and membrane blebbing [197, 198]. Oikonomou et al. discovered a novel role of DAPK1 in affecting the LC3-associated phagocytosis (LAP, also known as noncanonical autophagy) in fungal infection-related inflammation by modulating the formation of autophagosomes [199]. Interestingly, the LAP is an essential part of microglial phagocytosis in the brain. Chen et al. demonstrated that DAPK1 is also involved in the regulation of LAP in the microglia using an in vitro subarachnoid hemorrhage model by exposing microglial cells to oxyhemoglobin [200]. Oxyhemoglobin suppresses microglial LAP by upregulating p38 and downregulating DAPK1, further resulting in a reduction of cellular Beclin1 expression [200]. These data concomitantly support a crucial role of DAPK1 in cellular autophagy. Nevertheless, whether DAPK1 dysregulation is associated with autophagy impairment in AD is yet unresolved. Only one study reported that Aβ treatment activates neuronal DAPK1 and triggers autophagosome formation and apoptosis sequentially in primary neurons [156]. Importantly, neurons with high amounts of autophagosomes are devoid of apoptotic phenotypes [156], which suggests that the DAPK1-inducced autophagy might confer protection in AD. Taken together, the function of DAPK1 in microglia appears to be contradictory, as DAPK1 activation may stimulate inflammatory response, but it may also enhance autophagy to maintain cellular homeostasis.

## DAPK1 as a target for disease-modifying treatment of AD

Given the multiple roles of DAPK1 dysregulation in the pathogenesis of AD, DAPK1 has been an emerging target for the development of novel disease-modifying therapies for AD. Several types of molecule, including synthetic compounds, natural product-derived chemicals and peptide-based modulators have been tested in mouse models to evaluate the therapeutic potential of DAPK1 inhibition in various neuropathologies [201].

### Synthetic small-molecule DAPK1 modulators

The first small-molecule DAPK1 inhibitor was identified by screening a library of compounds, leading to the discovery of an aminopyridazine with a weak DAPK1 inhibitory effect [202]. Further structural optimization of the lead compounds resulted in the generation of two 3-amino-6-phenylpyridazine derivatives with remarkably improved inhibition and selectivity for DAPK1 (IC_50_ =  ~ 15 μM) [202]. These compounds have a common 6-phenyl group and competitively bind to the ATP-binding pocket of DAPK1. An in vivo study using a hypoxic ischemia rat model demonstrated that a single intraperitoneal injection of the candidate compound successfully ameliorated brain weight loss and preserved microtubule-associated proteins in the brain [202].

Okamoto et al. performed a structure-based virtual screening using the structures of target protein kinases, and identified a series of compounds with a common scaffold of 2-phenyl-4-(3-pyridinylmethylene)-5(4H)-oxazolone [203]. The pyridinyl group, the oxazolone ring and the phenyl ring in the scaffold are all essential for the inhibition of DAPK1 and DAPK3 activities based on the structure–activity relationship analysis [203]. More importantly, compounds with this scaffold are highly selective for DAPKs, facilitating the discovery of molecules with improved affinity. To discover novel DAPK1 inhibitors, Okamoto et al. carried out a multiple-step virtual screening, in which they first generated several binding-site models using molecular dynamics simulation to select structures with the highest enrichment, and then large-scale screening was performed based on the enriched DAPK1 structure [204]. Following the discovery of the lead compound, in vitro kinase assays and similarity search of more potent compounds were performed to obtain the optimal chemicals. This led to the discovery of 4-(pyridin-3-ylmethylene)oxazol-5(4H)-one (C6) [204], a highly efficient inhibitor for DAPK1 and DAPK3 (IC_50_ = 69 and 225 nM, respectively). This compound has been widely used in animal studies due to its strong inhibitory effect and high specificity for DAPK1. For example, we have shown that C6 is capable of reducing the secretion of Aβ40 and Aβ42 in the low nanomolar range [78]. Besides, C6 effectively prevents the ceramide- and Aβ aggregate-induced neuronal cell death [168]. In an epileptic mouse model, C6 treatment protected against the PTZ-induced seizures by diminishing the DAPK1-induced GluN2B phosphorylation [76].

Feng et al. designed a group of octahedral pyridocarbazole metal complexes based on the structure of the pan-kinase inhibitor Staurosporine [205]. The specific inhibitor OS4 contains ruthenium in the 1,4,7-trithiocycloalkane scaffold. Crystallographic analysis revealed extensive interactions between OS4 and the catalytic domain of DAPK1, including hydrogen bonds between the maleimide moiety and the hinge region of DAPK1, hydrophobic interactions between the 1,4,7-trithiacyclononane ligand group and the ATP-binding site and glycine-rich loop of DAPK1, and the occupation of the pyridocarbazole heterocycle in the adenine pocket of DAPK1 [205]. The IC_50_ values of OS4 for DAPK1 and DAPK3 are 2 and 8.8 nM, respectively. In addition, it preferentially inhibited DAPK1 activity with a selective factor higher than 4.4 in the kinase screening assay [205].

Carlson et al. applied a fluorescence-linked enzyme chemoproteomic strategy and found that HS38, a selective pyrazolo[3,4-d]pyrimidinone, can potently suppress DAPK1 and DAPK3 with an IC_50_ of about 200 nM [206]. The inhibition of DAPK3 by HS38 leads to reduced contractile force in mouse aorta and calyculin A-stimulated arterial muscle via decreasing the phosphorylation of regulatory myosin light chain (RLC20) [206]. Nevertheless, it is not clear how this compound regulates DAPK1 activity in ischemic stroke or other neurological disease models.

Singh et al. conducted virtual screening and molecular simulations to identify compounds that can simultaneously occupy the ATP-binding site and the substrate recognition site of DAPK1, which led to the discovery of the compound 11 as a promising inhibitor that directly interacts with the Glu100 and Glu143 residues of DAPK1 [207]. Furthermore, the prediction of drug-likeness also showed higher druggability and safety profile of the compound 11 than other compounds [207].

To target neuronal tauopathy, Farag et al. designed a dual inhibitor for DAPK1 and colony-stimulating factor 1 receptor (CSF1R) with a 3,5-dimethoxy-N-(4-(4-methoxyphenoxy)-2-((6-morpholinopyridin-3-yl)amino)pyrimidin-5-yl)benzamide structure [208]. This compound suppresses DAPK1 activity in a non-ATP competitive manner, as it binds to the substrate-binding site rather than the ATP-binding pocket of DAPK1. The IC_50_ value slightly decreased from 2.89 to 0.92 μM with the increase of ATP concentration in the in vitro kinase assay [208]. In addition, it also strongly inhibits the activity of CSF1R by competing with ATP. In vitro evaluation proved that the compound efficiently suppresses tau aggregation and lipopolysaccharide-induced neuroinflammation [208]. This compound is also highly selective toward DAPK1, despite the sequence homology between DAPK1 and other DAPK family members [208]. Moreover, this molecule might show encouraging neuroprotective effects due to its high BBB permeability and brain bioavailability [208].

Recently, rational design and synthesis of aryl carboxamide derivatives have led to the generation of an isonicotinamide derivative 4q as a promising DAPK1 inhibitor with an IC_50_ value of 1.09 μM [209]. The compound has been tested for anti-cancer activity in leukemia and breast cancer, while its effects on neuropathologies remain to be investigated [209]. Similarly, some 6,8,9-poly-substituted purine derivatives have been able to prevent leukemia cell proliferation and induce apoptosis by suppressing DAPK1 activity. Further structure optimization led to the discovery of 6-benzyloxy-9-tert-butyl-8-phenyl-9H-purine (compound 6d), an efficient DAPK1 inhibitor with an IC_50_ value of 2.5 μM [210]. Wilbek et al. performed high-throughput screening of novel DAPK1 inhibitors by applying a microfluidic capillary electrophoresis assay based on the DAPK1-induced specific phosphorylation of the Ser1303 residue of DAPK1 [211]. The identified compound (CPR005231) binds to the ATP pocket of DAPK1 at nanomolar concentrations and has an IC_50_ of 247 nM for DAPK1 [211]. However, this compound also inhibits the protein-tyrosine kinase Src at a similar IC_50_ [211]. Firoz et al. performed an in silico study aimed at obtaining DAPK1 inhibitors for the treatment of retinal degeneration, as studies suggested that DAPK1 contributes to ophthalmic disorders in large part by regulating the programmed cell death. They found several caspase inhibitor analogs with good inhibitory effects and promising drug-likeness properties [212].

In conclusion, a number of in silico studies have been performed to discover specific DAPK1 inhibitors, and indeed compounds with different scaffolds have been identified and tested in in vitro kinase assays. Although many studies usually include drug-likeness prediction which could provide some hints for further in vivo evaluation, animal experiments are urgently needed to better determine the effectiveness, pharmacokinetics and safety profiles of these small-molecule DAPK1 inhibitors.

### Natural compounds as DAPK1 modulators

Natural compounds represent an important resource for the discovery of therapeutic chemicals for various neurological diseases. Flavonoids are widely distributed bioactive molecules in nature and possess diverse biological effects such as anti-oxidative stress and anti-inflammation by interacting with key signaling molecules in the cell [213]. Yokoyama et al. first measured the binding affinity of 17 natural flavonoids to DAPK1 using the 1-anilinonaphthalene-8-sulfonic acid (ANS) competitive assay. Of all the tested flavonoids, morin, a natural polyphenol abundantly found in *Moraceae* species, showed the highest binding affinity toward the DAPK1 catalytic domain (IC_50_ = 1.6 ± 0.27 µM) in the ANS assay [214]. Crystallographic analysis revealed that these flavonoids mainly bind to the ATP pocket of DAPK1. In particular, it has been found that the 2’–OH of morin forms strong ionic interaction with the K42 residue of DAPK1, which underlies the robust inhibitory effect of morin on DAPK1 activity.

Resveratrol is a polyphenol ubiquitously found in grapes. It is a well-known dietary supplement due to its cardiovascular protective activity. Resveratrol directly binds to the ATP pocket of DAPK1 with an apparent dissociation constant of 8.5 μM [215, 216]. Specifically, the phenyl ring (A-ring) of resveratrol anchors at the adenyl base of ATP and the 3-OH group forms hydrogen bonds with the hinge region of DAPK1 [215]. Based on the binding mode, Yokoyama et al. further characterized several resveratrol derivatives using conventional biophysical approaches. Thermodynamic analysis of the binding suggested that the interaction between resveratrol derivatives and DAPK1 is mainly driven by favorable enthalpy changes due to the formation of hydrogen bonds and van der Waals interactions [216]. These derivatives all dock to the ATP pocket of DAPK1, and additional hydrogen bonds can be formed in the hinge region of DAPK1 [216]. A similar binding mode was reported for Purpurin, an anthraquinone isolated from the roots of *Rubia cordifolia*, for its interaction with DAPK1 [217]. The IC_50_ value of Purpurin for DAPK1 was determined to be 890 nM [217]. Talwar et al. carried out virtual screening in combination with in vitro kinase assay to discover DAPK1 inhibitors from the ZINC database [218]. The compound-N4 binds to the ATP pocket and the substrate recognition site of DAPK1 [218].

Quercetin is a ubiquitously distributed BBB-permeable flavonoid. Qi et al. identified DAPK1 as a downstream target of Quercetin using a systematic approach [219]. Very interestingly, they observed that HT-22 cells treated with Aβ species showed an increase in DAPK1 mRNA expression which is consistent with their microarray analysis of the GSE5281 dataset, and Quercetin treatment dramatically suppressed this increase [219]. This finding indicates that Quercetin is likely beneficial for ischemic stroke and AD by indirectly targeting DAPK1. In all, although biophysical and crystallographic data support the idea that these natural compounds directly bind to DAPK1, it remains elusive whether these molecules indeed exert protective effects through inhibiting DAPK1 in vitro and in vivo.

There are also natural compounds that can upregulate DAPK1 function in cells. For instance, Sanggenon C is able to decrease the expression of the E3 ubiquitin ligase Mind-bomb 1 (Mib1, also known as DIP-1), an important binding partner controlling the degradation of DAPK1 in the cell [52, 53]. Sanggenon C upregulates the cellular DAPK1 level by stabilizing the protein, leading to cell cycle arrest and apoptosis in glioblastoma model [220]. Grifolin has been reported to upregulate the cellular p53 function and promote the transcription and expression of DAPK1 in breast cancer cell lines, thus promoting cancer cell death [221]. Curcumin has also been shown to cause cell cycle arrest and apoptosis in cancer cells by upregulating DAPK1 transcription and its protein level [222]. Notably, these data are mostly obtained using cancer cell lines and it is not clear whether these molecules also show similar effects in neuronal cells.

### Peptide-based DAPK1 modulators

Many of the biological functions of DAPK1 are mediated by its cellular substrates. Specific amino acids or sequences involved in the recognition and binding events can therefore be utilized to design peptide-based blockers to interfere with DAPK1 dysregulation-induced pathological changes. This method has several advantages over small-molecule inhibitors. First, peptide-based blockers are highly selective and specific toward the protein targets and their substrates. Second, peptide-based blockers can be easily modified to increase the inhibition potency and biological diversity [223]. Third, the metabolism of peptides generates amino acids with low cellular toxicities [224]. Nevertheless, peptide-based compounds are susceptible to rapid proteolysis and insufficient membrane penetration in vivo, which hinder their translational applications in neurological diseases [225]. In this part, we will discuss the use of blocking peptides as a tool to specifically disrupt DAPK1–substrate interactions for disease intervention.

The blocking peptide approach was initially employed by Tu et al. to block the interaction between DAPK1 and GluN2B in an ischemic stroke mouse model [73]. In their study, the direct binding sequence of GluN2B (1292–1304 aa) was fused to the cell-membrane transduction domain of the HIV-1 Tat protein (Tat-NR2B_CT_) [73]. This peptide completely abolished the interaction between DAPK1 and GluN2B in neurons, thereby resulting in reduced GluN2B phosphorylation and subsequent Ca^2+^ influx in mouse brain [73]. Furthermore, the Tat-NR2B_CT_ treatment was sufficient to improve ischemic stroke-induced brain infarction and chronic stress-induced depressive-like behaviors in vivo [73, 109]. The same group also identified a minimal binding sequence in the DNA-binding motif of p53 that is responsible for the interaction with the death domain of DAPK1. The fragment was conjugated with the cell-membrane transduction domain of Tat protein to generate a blocking peptide Tat-p53DM, which has been shown to suppress DAPK1-induced p53 phosphorylation in cortical neurons subjected to oxygen–glucose deprivation [101]. Moreover, intravenous injection of the peptide not only rescued brain infarction and neuronal cell death, but also reversed motor deficits in ischemic stroke mouse model [150]. The interaction between DAPK1 and tau can also be prevented by using a membrane-permeable peptide containing the minimal tau-binding sequence [100]. The peptide can cross the BBB when administered intravenously. It is capable of ameliorating stroke-induced dendritic spine damage and cognitive injuries by attenuating the DAPK1-induced tau phosphorylation in neurons [100]. Inspired by this, our group also developed a specific blocking peptide (Tat-DM) for the interaction between ERK and DAPK1. This peptide interrupts ERK-induced DAPK1 phosphorylation at Ser735 by uncoupling the binding between these two proteins, thus inhibiting the catalytic activity of DAPK1 [77]. Treatment of Tat-DM successfully reduced neuronal apoptosis and suppressed seizure severity in KA-induced mouse epilepsy model in vivo by downregulating DAPK1 activity in the brain [77]. These data together demonstrate the promising application of peptide-based DAPK1 modulators in the intervention of neurological diseases. They are not only robust tools for research purposes, but also possess translational potential for clinical applications. However, peptides usually suffer from low biostability and poor BBB permeability in vivo, which strongly limit the bioavailability and efficacy of peptide-based therapeutics in human brains [226]. Strategies such as peptidomimetics, chemical modifications and bionanotechnology-based delivery systems might be applied to overcome these drawbacks [223].

### Perspectives regarding the development of DAPK1 modulators

Protein kinases are valuable therapeutic targets for the intervention of AD, not only due to the direct participation of dysregulated kinases in various AD pathologies, but also because of the vast number of chemicals and strategies available for drug development [227]. Compounds targeting GSK-3β, CDK5, p38 MAPK, ERK1/2, JNK3 or several tyrosine kinases have been evaluated in AD mouse models, and some of them have reached clinical trials [228]. For instance, short-term (10 weeks) treatment of lithium, a GSK-3β inhibitor, had no impact on CSF biomarkers and cognitive performance in AD patients. However, a prolonged treatment of lithium (over 12 months) in patients with mild cognitive impairment led to improved cognitive function and reduced tau phosphorylation with favorable safety profiles [229, 230]. Tideglusib, another GSK-3β inhibitor that has been evaluated in a 26-week, multicenter phase II clinical trial, also demonstrated positive effects on cognitive performance in patients with mild AD [231]. Other kinase inhibitors such as Neflamapimod for p38 MAPK and NE3107 for ERK1/2 are being analyzed in AD patients and have shown promising results in phase II clinical trials [227]. However, CDK5 or JNK3 inhibitors are less investigated in clinical trials although these kinases play a pivotal role in the pathogenesis of AD. CDK5 and JNK3 are crucial for cellular signaling transduction, meaning that non-selective suppression of the activities of the kinases may induce toxic rather than therapeutic effects in AD patients [232].

The extensive involvement of DAPK1 in regulating multiple AD pathological changes makes it an ideal target for the development of disease-modifying treatments for AD. Indeed, several synthetic DAPK1 inhibitors and blocking peptides have already been exploited in AD or stroke mouse models for disease intervention. The neuroprotective effect of DAPK1 inhibition on ischemic stroke has been systematically summarized by Khan et al. [233]. In their study, they compared the effects of different approaches in inhibiting neuronal DAPK1 function, including gene knockout, post-transcriptional silencing by miRNAs, kinase inhibitors and blocking peptides, and concluded that post-transcriptional regulators and blocking peptides provide better functional outcomes than other methods [233]. However, it remains to be determined whether this also applies to chronic neurodegenerative diseases such as AD. Besides, structure-based virtual screening and high-throughput screening are continuously conducted to identify new molecular entities with potent inhibitory effect and high selectivity for DAPK1. Nevertheless, most of the compounds are only evaluated for the IC_50_ values and binding affinities using in vitro assays. Whether the candidate compounds show direct target engagement in animal models remains to be studied.

Although several DAPK1 inhibitors have been evaluated in animal models and show promising neuroprotective effects on ischemic stroke and epilepsy [234], the pharmacological profiles of these candidates are yet to be systematically evaluated. Besides, whether the lead compounds are effective in improving neuropathological changes and behavioral performance in chronic disease models such as AD and PD remains uncharacterized. Moreover, none of the aforementioned DAPK1 modulators have been tested in clinical trials to date [201]. Based on the lessons from clinical research on other kinase inhibitors, we propose several key points in the development of DAPK1 modulators for disease-modifying treatment of AD. First, *DAPK1* is an important tumor suppressor gene that is downregulated in a wide range of cancers [235]. Non-selective inhibition of DAPK1 in tissues without DAPK1 dysregulation may cause unwanted side effects, which highlights the necessity of designing brain-specific DAPK1 inhibitors for disease intervention. Second, the BBB may limit the penetration of chemicals into the brain parenchyma, thus reducing the effective concentration of the compound in the brain. Therefore, increasing the BBB permeability to of compounds is an important aspect in compound screening and structural optimization. Third, the currently available DAPK1 inhibitors mostly bind to the ATP pocket of DAPK1 in the kinase domain, which is highly homologous among DAPK family proteins. It would be helpful to design molecules with additional interacting patterns, such as the substrate recognition motif to obtain more selective and specific inhibitors. This could also avoid the undesirable inhibition of other types of protein kinases in the cell. In all, given that the neuropathologies in AD generally require long-term interventions, DAPK1 modulators with balanced efficacy, safety property and favorable pharmacological profiles should be developed for further preclinical and clinical studies.

## Conclusions

AD is an ageing-related progressive neurodegenerative disorder. The Ca^2+^/CaM-dependent Ser/Thr kinase DAPK1 has been implicated in neuronal loss in ischemic stroke, TBI and neurodegenerative diseases such as AD. Several lines of evidence demonstrate that DAPK1 is a promising therapeutic target for the disease-modifying treatment of AD. First, DAPK1 is highly expressed in adult hippocampus, a brain region that is particularly vulnerable to Aβ and tau pathologies during AD progression. Our research has also established a causal link between DAPK1 dysregulation and core AD neuropathologies, supporting DAPK1 as a common upstream regulator of Aβ generation and tau hyperphosphorylation in AD. Second, in addition to Aβ and tau pathologies, DAPK1 has been suggested to affect neuronal cell death and synaptic function in the brain, two events that are directly connected with the structural and functional integrity of the brain. The multifunctional role of DAPK1 is closely associated with different neuropathological changes in AD, making it possible to achieve comprehensive treatment by targeting one protein. Third, there are already synthetic and natural product-derived compounds available for further identification and evaluation of DAPK1 inhibitors. Moreover, some compounds have been assessed in vivo and shown promising effects in AD mouse models, which could offer additional insights into the design and optimization of molecules with improved efficacy and safety profiles. Additionally, to better understand the exact contribution of DAPK1 dysregulation to the pathophysiological changes of AD, several key issues need to be solved. For example, how do the expression and activity of DAPK1 change along the disease progression of AD? Can we develop DAPK1 as an early diagnostic marker for the clinical assessment of AD progression? Does DAPK1 interact with other AD risk factors such as ApoE and TREM2 in the pathogenesis of AD and how? What is the pathophysiological role of DAPK1 in glial cells and how is this associated with AD pathologies? We believe that answering these questions is as important as the development of specific DAPK1 modulators for disease intervention, and will definitely deepen our knowledge about the physiological and pathological roles of DAPK1 in the brain.

## Data Availability

Not applicable.

## Abbreviations

AD: Alzheimer’s disease
Aβ: Amyloid-β
DAPK1: Death-associated protein kinase 1
PD: Parkinson’s disease
NFT: Neurofibrillary tangle
BBB: Blood–brain barrier
CSF: Cerebrospinal fluid
APP: Amyloid precursor protein
PS1: Presenilin-1
LOAD: Late-onset AD
DRP-1/DAPK2: DAPK1-related protein 1
ZIPK/DAPK3: Zipper-interacting protein kinase
DRAK1: DAPK1-related apoptosis-inducing protein kinase 1
CaM: Calmodulin
ROC: Ras of complex
COR: C-terminal of ROC
HSP90: Heat shock protein 90
DIP-1: DAPK-interacting protein 1
Pin1: Peptidyl prolyl isomerase 1
DD: Death domain
TBI: Traumatic brain injury
NMDAR: N-methyl-D-aspartate receptor
PTZ: Pentylenetetrazol
MPTP: 1-Methyl-4-phenyl-1,2,3,6-tetrahydropyridine
GWAS: Genome-wide association study
SNP: Single-nucleotide polymorphism
KA: Kainic acid
CREB1: CAMP response element-binding protein 1
PP2A: Protein phosphatase 2A
MARK2: Microtubule affinity-regulating kinase 2
IL-1β: Interleukin-1β
NDRG2: N-myc downstream-regulated gene 2
UNC5C: Uncoordinated-5 homologue C
LTP: Long-term potentiation
LTD: Long-term depression
ECII_*PN*_: Pyramidal neurons in the entorhinal cortical layer II region
α-syn: α-synuclein
NLRP3: NOD-, LRR- and pyrin domain-containing 3
LAP: LC3-associated phagocytosis
CSF1R: Colony-stimulating factor 1 receptor
ANS: 1-Anilinonaphthalene-8-sulfonic acid
Cyp D: Cyclophilin D
